# Supplementary material for: Standing-up against moral violations: The predicting role of attribution, kinship, and severity
Source: PLoS One. 2024 Aug 12;19(8):e0307740. doi: 10.1371/journal.pone.0307740 (PMC11318881; doi:10.1371/journal.pone.0307740)
Supplement: Attachment — Submitted filename: Comments.docx [file pone.0307740.s001.docx]

**Comments**

Abstract: Overall, the abstract effectively summarizes the main points of the research and its findings. It clearly states the research question and provides an overview of the hypotheses and predictions. However, some aspects could be further improved:

Lack of Methodological Information

Generalizability and External Validity

Limited Discussion of Implications

Incomplete Evidence on Costs of Intervention

Introduction:

The introduction lacks a clear and concise research question or hypothesis. While it mentions the aim of investigating the predicting functions of responsibility attributions, kinship, and severity on people's willingness to intervene, it does not explicitly state the specific research question that the study aims to answer. Clearly state the research question or hypothesis that the study aims to address. This will provide a clear focus for the introduction and guide the reader's understanding of the study's objectives.

The organization of the introduction is somewhat disjointed. The transition between different topics and concepts is not always smooth, and the flow of the text could be improved. Refine the organization and flow of the introduction. Ensure that there is a logical progression of ideas and that transitions between concepts are smooth.

The introduction would benefit from a clearer explanation of the theoretical frameworks (attribution theory and kinship theory) and their relevance to the study. The current explanation is brief and may leave readers wanting more context. Provide a more comprehensive explanation of the theoretical frameworks (attribution theory and kinship theory) and their applicability to the study. This will help readers understand the theoretical foundations of the research and how they relate to the specific research question.

Methodology and Results:

The study presents an investigation into the effects of attribution, situational attributes, and kinship on helping behavior. The authors provide a detailed description of their methodology, including the power analysis, participants, design, procedures, and dependent variables. The results are reported, including main effects and interactions. Overall, the study appears to be well-designed and the results are presented clearly. However, there are some areas that require further clarification and improvement. The following critical analysis addresses these issues;

Power Analysis and Sample Size: The authors state that they aimed to detect a small effect size (η² = .05) with a power of .80. However, the power analysis using GPower indicated a required sample size of 249 participants, while the actual number of participants tested was 290. This raises questions about the accuracy of the power analysis and whether the sample size was determined appropriately. The authors should provide a clear explanation or justification for the larger sample size.

Ethical Approval: The authors state that according to the University of Regensburg's ethic committee, no ethical approval was needed for the experiment. It is essential to provide more information regarding the criteria used by the ethics committee to determine that ethical approval was not necessary. This clarification is important to ensure that the study was conducted ethically and in line with ethical standards.

Random Assignment: The authors mention that participants were randomly assigned to one of the 12 experimental conditions. However, it is not clear how the randomization was performed. Providing details about the randomization procedure would enhance the transparency and rigor of the study.

Manipulation Check: The manipulation check results indicate a significant difference between high and low responsibility conditions, supporting the success of the responsibility manipulation. However, the effect size reported (d = 1.56) appears to be quite large. Such a large effect size raises concerns about the robustness of the manipulation. The authors should discuss potential limitations or alternative explanations for such a strong effect.

Marginal Effect: The authors report a marginal effect for kinship (F(1, 278) = 3.58, p = .059, ω² = .01). While the effect did not reach conventional levels of statistical significance, it is still important to interpret and discuss this finding in the context of the research question. The authors should provide a more thorough analysis and discuss potential implications of this marginal effect.

Figure 1 and Error Bars: Figure 1 presents behavioral intentions to help a person in need, but the error bars representing the 95% confidence intervals are not clearly defined. It is crucial to provide a clear explanation of the error bars, including how they were calculated and what they represent. Additionally, the figure should be labeled appropriately to provide a clear understanding of the data being presented.

Here are some deficiencies or areas that could be improved in the general Discussion section:

Lack of Quantitative Results: The section primarily focuses on summarizing the findings and interpreting them in relation to existing theories. However, it lacks specific quantitative results, such as effect sizes or statistical significance levels. Including these details would provide a more precise understanding of the findings and increase the rigor of the discussion.

Incomplete Explanation of Responsibility-Based Effects: The section mentions that responsibility-based effects were not observed in more-severe situations, but it does not delve into the possible reasons for this discrepancy. Exploring potential explanations or discussing alternative theoretical frameworks that could account for the lack of responsibility effects in severe situations would enhance the analysis.

Insufficient Explanation of Kinship Theory Findings: Although the authors acknowledge the absence of evidence for kinship theory as a predictor, the provided explanations for this discrepancy appear somewhat speculative and lack substantial empirical support. Providing a more in-depth analysis of the potential reasons for the deviation from previous research and discussing alternative explanations would strengthen this part of the discussion.

Lack of Discussion on Sample Representativeness: The section does not mention the characteristics of the sample or any potential limitations related to sample representativeness. Including information about the demographics and characteristics of the participants, as well as addressing any potential biases or limitations associated with the sample, would add depth and context to the study's findings.

Limited Generalizability of the Findings: The section does not explicitly address the generalizability of the findings beyond the specific experimental context used in the study. Recognizing the limitations of generalizability and discussing the potential boundary conditions or contexts where the findings may not hold would provide a more comprehensive understanding of the research implications.
