# Supplementary material for: Standing-up against moral violations: The predicting role of attribution, kinship, and severity
Source: PLoS One. 2024 Aug 12;19(8):e0307740. doi: 10.1371/journal.pone.0307740 (PMC11318881; doi:10.1371/journal.pone.0307740)
Supplement: Attachment — Submitted filename: Response to Reviewers.docx [file pone.0307740.s002.docx]

**Editorial Comments:**

**E1:** “PLOS ONE's style requirements”

Please ensure that your manuscript meets PLOS ONE's style requirements, including those for file naming.

RESPONSE:

Thank you for pointing this out. In accordance with PLOS ONE's style requirements, we changed the style of the headings in the manuscript and the title page’s layout. Additionally, the files are now named: Response to Reviewers, Revised Manuscript with Track Changes, and Manuscript.

**E2:** “Gender nouns”

Please change "female” or "male" to "woman” or "man" as appropriate, when used as a noun.

RESPONSE:

Thank you very much for this important remark. We changed all nouns according to APA’s guidelines.

**E3:** “Data Availability”

We note that you have stated that you will provide repository information for your data at acceptance. Should your manuscript be accepted for publication, we will hold it until you provide the relevant accession numbers or DOIs necessary to access your data. If you

wish to make changes to your Data Availability statement, please describe these changes in your cover letter and we will update your Data Availability statement to reflect the information you provide.

RESPONSE:

Thank you for pointing this out. As soon as the MS should be accepted, we change the OSF-repository from “View-only” to “public”. The data availability statement will then read: “All data, materials, and R-scripts are available at the OSF platform (<https://osf.io/8bfe2>, DOI 10.17605/OSF.IO/8BFE2) .”

# Reviewer #1:

*Major Concerns:*

**R1.1:** “The submitted manuscript presents data investigating the effects of severity of outcome, responsibility for outcome, and kinship on self-reported willingness to intervene in response to hypothetical moral scenarios. The submitted manuscript presents findings across

two studies providing support for the perceived responsibility and severity of outcome as two crucial predictors of an individual’s self-reportedratings of willingness to intervene. While this work is important, the current manuscript is difficult to parse as it relates to the

stated study aims, experimental design, and analytic approach. I have outlined specific comments below.”

RESPONSE:

First of all, we would like to thank you for your very helpful and constructive feedback. We address each of your comments below.

**R1.2:** "The introduction states the importance of differentiating between moral courage and other forms of helping behaviors at length. As such, the ability to experimentally investigate independent effects of these hypothesized distinct forms of helping is central in the current form of the manuscript. However, in the current experimental design, moral courage is conflated with severity and is modeled in the analyses as a moderate level in the severity factor. As such, it is impossible for conclusions to be drawn regarding specificity of

moral courage. It is unclear how essential this is to the study design as the hypotheses seem predominantly concerned with severity, responsibility, and kinship. If this is the case, the authors should adjust the discussion of moral courage and remove conclusions specific to moral courage and focus instead on severity of outcome.”

RESPONSE:

Thank you very much for this recommendation. Absolutely, we agree with you that the moral courage situations we used might be conflated with severity. However, all helping situations are, at least partly, a function of severity. Given that our pattern of results is stable, and that we used moral courage scenarios that have been successfully used in the existing literature (e.g., Fischer et al., 2006; Greitemeiyer et al., 2003, Sasse et al. 2022), we are optimistic that our findings help to better understand whether people will act morally courageous. Nonetheless, we elaborated on the potential conflation of severity (p. 21, line 495). “Nevertheless, we are aware of that moral courage situations vary in severity. Therefore, severity might interfere with the specific of moral courage situations. However, the pattern of results remained across our studies. Given that the used moral courage situations were might more severe than others, we highly encourage upcoming research to further explore whether our findings expand to less severe moral courage situations (9)“.

Moreover, we better linked our findings with moral courage wherever possible. Additionally, we shortened our explanation concerning moral courage.

**R1.3: “**Related – the lack of consistency across study 1 & 2 with regard to the “moral courage” scenario make it even more difficult to assert empirical investigation of moral courage. The authors do not provide much justification for why these violations specifically represent instances of moral courage. Furthermore, including only one scenario per category makes the specific details of the scenarios even more important as idiosyncrasies of the scenarios may be driving the effects (ie. violating political ideology vs. sexual harassment). This is stated in a roundabout way in the discussion section but should be further elaborated.”

RESPONSE:

Thank you for pointing this out. We totally agree with you that it is important to vary stimuli, especially following the Brunswikian idea of systematic and representative research designs. Given that the existing literature has shown quite stable effects for low-severe and, respectively, high-severe situations, we opted to only vary the moral courage scenario to examine whether our predictions generalize. We emphasized this argument (p. 23, line 543): “Second, we only varied the moral courage scenarios; hence our sample of prosocial situations was rather limited. Moreover, we are aware of that prosociality in moral courage is complex and can occur in different facets (69). Thus, following the Brunswikian idea of systematic and representative research designs (80–82), we highly encourage future research to systematically vary situations to get a full-fledged understanding of the predictors of prosocial behavior (i.e., moral courage).”

**R1.4: “**The authors conduct full-factorial between subjects analyses in both studies including modeling the 3-way interaction. Given that there are no hypotheses related to the three-way hypothesis and there is unlikely to be sufficient power to detect a three-way hypothesis, I would suggest the authors remove the three-way interaction from their analyses. If the authors choose to keep the threeway interaction, hypotheses and support for this inclusion should be provided in the introduction.”

RESPONSE:

Thank you very much for this remark. Indeed, we did not predict a three-way-interaction, but we included the analysis for transparency. We emphasized this in results section to make this clear for potential readers. (p. 13, line 301): “Although we did not predict a *Severity* by *Responsibility* by *Kinship* interaction, we included the three-way test for transparency. The three-way-interaction was not significant, *F*(2, 278) = 0.78, *p* = .457.“ The formulation for study 2 is identical.

**R1.5: “**The mediation analysis in study 2 is interesting however the conclusion that it is the cost of helping underlying the effect isn’t entirely clear. The way the study is designed, one cannot rule out that it is the higher cost of NOT helping the person in need (ie. greater severity for the victim not the helper).”

RESPONSE:

Thank you very much for this insightful remark. We agree with you that costs for not helping are a potential mediator. Given that we conceptualized our studies on previous articles, and those did not include costs for not helping a person in need, we only focused on costs for helping. Consequently, we included this highly valuable point in our limitations. Thank you again for pointing this out. (p.23, line 532). “However, our results cannot rule out the potential mediating effect of costs for not helping. For example, you observe a toddler locked in a car during summer heat. In this scenario, your costs for acting are rather low (e.g., calling 911, or smashing a side window), while the costs for not helping are extremely high. Thus, we highly encourage future research to examine costs for not helping as an additional underlying psychological process. We want to thank an anonymous reviewer for this important point.“

*Minor Concerns:*

**R1.6:** “Study 1 & Study 2 differ dramatically based on sex and age of the study population. The authors should include a comparison of the two samples across demographic variables. Study 1 in particular is ~77% female – this should be acknowledged directly and addressed

in the discussion.”

RESPONSE:

Thank you very much for this comment. We agree with you that gender can potentially interfere with our findings (Eagly & Crowley, 1986). Consequently, we added a check for interfering effects for both studies.

(p. 12, line 272) “Previous research revealed that men are more likely to help than women (65). Consequently, we performed a log-linear analysis to check whether participants were equally distributed across conditions regarding their gender. The model indicated no significant difference concerning participants’ gender distribution across conditions, χ^2^ (29) = 13.99, p = . 991.”

(p. 17, line 391) “As in Study 1, we performed a log-linear analysis to check whether participants were equally distributed across conditions regarding their gender. The model indicated no significant difference concerning participants’ gender distribution across conditions, χ2 (18) = 11.52, p = . 871.”

Regarding age, previous research revealed rather mixed evidence concerning age as a predictor for pro-social behavior (e.g., Bailey et al., 2020; McAdams et al., 1993; Steblay, 1987; Sze et al., 2012). Most results showed that children become more pro-social with increasing age (e.g., Smith & Hard, 2002). For adults, older adults seem to be more pro-social than younger (e.g., McAdams et al., 1993, Sze et al., 2012). However, the definition of older adults is rather vague; older than 37 (McAdams et al., 1993) vs 60 to 80 (Sze a., 2012).

Nevertheless, we applied a linear model to check whether age predicted participants’ willingness to intervene. We included this additional information.

(p. 12, line 272) “Moreover, the existing literature provides, although somewhat mixed, evidence that age can predict people’s willingness to intervene. Therefore, we ran a linear regression to check whether participants’ age predicted their willingness to intervene. The results indicate that age did not significantly predict the willingness to intervene, *F*(284) = 0.30, *p* = .581.

(p. 17, line 393) “Analogously, we ran a linear regression to check whether participants’ age predicted their willingness to intervene. The results indicate that age did not significantly predict the willingness to intervene, *F*(344) = 0.48, *p* = .490.

Moreover, the pattern of our results remained the same after including age as a control variable. To keep our results section as concise as possible, we only refer to our additional analyses that can be retrieved from OSF.

Finally, we applied a *t*-test to check whether our studies differ concerning participants’ overall willingness to intervene. We added this information to our limitations. (p.23, line 548) “Third, some might argue that our samples differ concerning their demographics (i.e., age and gender), and that they are not representative. Although they might differ, we did not find a significant difference in their overall willingness to intervene, *t*(634) = 1.08, *p* = . 282, *d* = 0.09, 95*%* CI [-0.07, 0.24].”

**R1.7:** “The OSF link was not functioning to see the moral scenarios.”

RESPONSE:

Thank you for your comment and checking our material on OSF. We apologize for the inconvenience. We added a new OSF-link. You can find the moral courage scenarios in pdf named “4_mcacks_study_1_quest_eng.pdf” from p. 14 onwards.

**R1.8:** “Lines 300-303 and 394-397 should be describing the low-severity effects but it is unclear. Please revise the language.

RESPONSE:

Thank you very much for pointing this out. We rewrote both sections to improve readability. Please note that we only include one change here, because both sections are identical.

(p 13., line 294) “However, responsibility did not significantly affect people’s willingness to intervene in moral courage situations (not responsible: *M* = 5.68, *SD* = 1.21; responsible: *M* = 5.41, *SD* = 1.45), *t*(278) = 1.02, *p* = .312. Similar results could be overserved for more-severe situation (not responsible: *M* = 6.17, *SD* = 1.25; responsible: *M* = 6.41, *SD* = 0.72, *t*(278) = -0.91, *p* = .358).”

**R1.9:** “Lines 430-434 are confusingly written – is this asserting that the mediation was partial?”

RESPONSE:

Thank you very much for this remark. We now directly state that the mediation was partial. (p. 19, line 439): “However, the mediation analysis showed that costs for helping partially mediated participants’ willingness to intervene. In other words, the situation itself predicted the willingness to intervene. Precisely, participants in the moral courage condition were more willing to intervene compared to those in the less-severe helping condition (*c‘*_1_ = .0768). Moreover, participants in the more-severe condition were more willing to intervene compared to the less-severe helping condition (*c‘*_2_ = 0.457).”

**Reviewer #2:**

**R2.1: “**Abstract: Overall, the abstract effectively summarizes the main points of the research and its findings. It clearly states the research question and provides an overview of the hypotheses and predictions. However, some aspects could be further improved: Lack of Methodological Information, Generalizability and External Validity, Limited Discussion of Implications, Incomplete Evidence on Costs of Intervention”

RESPONSE:

First of all, we would like to thank you for your very helpful and constructive feedback, which substantially contributed to an improvement of our manuscript. Please note that, all page and line numbers refer to the “Manuscript” file.

*Introduction*

**R2.2:** “The introduction lacks a clear and concise research question or hypothesis. While it mentions the aim of investigating the predicting functions of responsibility attributions, kinship, and severity on people's willingness to intervene, it does not explicitly state the specific research question that the study aims to answer. Clearly state the research question or hypothesis that the study aims to address. This will provide a clear focus for the introduction and guide the reader's understanding of the study's objectives.”

RESPONSE:

Thank you very much for this remark. Our main research question was to assess whether, predictions derived from attribution and, respectively, evolutionary theory can be transferred to moral courage situations. We added this information to be clearer about our research question. (p. 7 , line 170). “In other words, we aim to answer the question whether predictions derived from attribution and, respectively, evolutionary theory can be transferred to moral courage situations.“

**R2.3:** “The organization of the introduction is somewhat disjointed. The transition between different topics and concepts is not always smooth, and the flow of the text could be improved. Refine the organization and flow of the introduction. Ensure that there is a logical

progression of ideas and that transitions between concepts are smooth.”

RESPONSE:

Thank you very much for this important suggestion. We heeded your suggestion and restructured our introduction to improve its natural flow and readability. Thank you again for this advice.

*Methodology and Results*

**R2.4** “The study presents an investigation into the effects of attribution, situational attributes, and kinship on helping behavior. The authors provide a detailed description of their methodology, including the power analysis, participants, design, procedures, and dependent

variables. The results are reported, including main effects and interactions. Overall, the study appears to be well-designed and the results are presented clearly. However, there are some areas that require further clarification and improvement. The following critical

analysis addresses these issues”

RESPONSE:

Thank you for your important comments. We address each point below.

**R2.5** “Power Analysis and Sample Size: The authors state that they aimed to detect a small effect size (η² = .05) with a power of .80. However, the power analysis using GPower indicated a required sample size of 249 participants, while the actual number of participants tested was 290. This raises questions about the accuracy of the power analysis and whether the sample size was determined appropriately. The authors should provide a clear explanation or justification for the larger sample size.”

RESPONSE:

Thank you very much for pointing this out. We tested more participants than the power analysis had indicated to compensate for potential attrition. Given that that attrition rates can vary from 0% to 80% (e.g., Weidman & Miratrix, 2021; Zhou & Fischbach, 2020), we opted to compensate for a minimum of 10% attrition (Rosenthal & Rosnow, 2009). (p. 10, line 225): “We factually tested 290 participants (223 women, 65 men, two participants did not indicate their sex), to compensate for a minimum attrition rate of 10% (63).”

**R2.6** “Ethical Approval: The authors state that according to the University of Regensburg's ethic committee, no ethical approval was needed for the experiment. It is essential to provide more information regarding the criteria used by the ethics committee to determine that ethical approval was not necessary. This clarification is important to ensure that the study was conducted ethically and in line with ethical standards.”

RESPONSE:

Thank you for this important remark. We totally agree with you that a clarification is important to ensure that our studies adhere to ethical standards. The University of Regensburg's ethic committee uses a short [checklist](https://www.uni-regensburg.de/assets/ethikkommission/ek-ur/Brauche_ich_ein_Ethikvotum_.pdf) to assess whether an ethical approval is needed for a research project. Given that our studies did not indicate that we need to apply for a full approval. We rewrote this section to make it clearer for potential readers and added the checklist to our OSF page. (p. 10, line 231). “In line with the University of Regensburg’s ethic regulations, we scrutinized our studies to assess whether a full ethical approval would be sensible. This assessment indicated that we did not need further ethical approval for our experiments.”

**R2.7** “Random Assignment: The authors mention that participants were randomly assigned to one of the 12 experimental conditions. However, it is not clear how the randomization was performed. Providing details about the randomization procedure would enhance the

transparency and rigor of the study.”

RESPONSE:

Thank you for this important recommendation. We ensured randomization by applying R’s sample()-function, which uses stochastic simulations (Ripley, 1987). Heeding your recommendation, we added this information for study 1 and study 2. (p. 11, line 238; p. 15, line 359). “We used R’s sample()-function to randomly assign participants to one of the twelve experimental conditions. The R-code can be retrieved from our OSF page.”

**R2.8:** “Manipulation Check: The manipulation check results indicate a significant difference between high and low responsibility conditions, supporting the success of the responsibility manipulation. However, the effect size reported (d = 1.56) appears to be quite large. Such a

large effect size raises concerns about the robustness of the manipulation. The authors should discuss potential limitations or alternative explanations for such a strong effect.”

RESPONSE:

Thank you very much for this remark. We totally agree with you that a Cohen’s *d* of 1.56 might seem extraordinarily large. However, compared to preceding literature that used a similar manipulation (Greitemeyer et al., 2003), our effect was smaller. We recalculated the effect size from Greitemeyer et al (2003), which turned out to be a Cohen’s *d* of 2.63. For further precision and transparency, we added the recalculated effect size and the CIs of our *d* to the methods section of Study 1 and Study 2 (p. 12, line 273). “… *d* = 1.56, 95*%* CI [1.30, 1.83]. For transparency, we recalculated the effect size of previous research (51) that revealed a Cohen’s *d* = 2.63, hence we state that we found a smaller effect size. Nevertheless, we may thus say that the responsibility manipulation was successful.” We used the same wording for study 2.

**R2.9:** “Marginal Effect: The authors report a marginal effect for kinship (F(1, 278) = 3.58, p = .059, ω² = .01). While the effect did not reach conventional levels of statistical significance, it is still important to interpret and discuss this finding in the context of the research question. The authors should provide a more thorough analysis and discuss potential implications of this marginal effect.

RESPONSE:

Thank you very much for pointing this out. Indeed, we only found a marginal effect for kinship in study 1. We heeded your recommendation, an provided an elaborated explanation why we did not find an effect for kinship. (p. 22, line 521) “Fourth, although we did not assess emotional closeness, emotional closeness has been shown to account for a substantial portion of the effect of genetic relatedness on the willingness to act pro-social (68). Thus, we encourage future research to assess emotional closeness to disentangle kinship from social closeness effects.“

Additionally, we tested the effect for kinship across our two studies. We did not find a significant effect for kinship on willingness to intervene, *t*(634) = 1.64, *p* = .100, *d* = 0.13, 95*%* CI [-0.03, 0.29]. To keep the manuscript as concise as possible, we included this in the supplements (R-script with additional analyses).

**R2.10:** “Figure 1 and Error Bars: Figure 1 presents behavioral intentions to help a person in need, but the error bars representing the 95% confidence intervals are not clearly defined. It is crucial to provide a clear explanation of the error bars, including how they were calculated and what they represent. Additionally, the figure should be labeled appropriately to provide a clear understanding of the data being presented.”

RESPONSE:

Thank you for this important remark. We changed the title of our figures to “Self-reported intentions to help a person in need”, to improve readers’ understanding. Additionally, we added that the confidence intervals represent the 95% CIs of the mean, and that we calculated them following Cohen et al.’s (2003) recommendation.

The figure captions (including titles) now read as follows: “*Fig. 1* Behavioral intentions to help a person in need. Caption. Error bars represent 95% confidence intervals of the mean. We calculated the confidence intervals as recommended by (63).”

*General discussion*

**R2.11:** “Lack of Quantitative Results: The section primarily focuses on summarizing the findings and interpreting them in relation to existing theories. However, it lacks specific quantitative results, such as effect sizes or statistical significance levels. Including these details would provide a more precise understanding of the findings and increase the rigor of the discussion.”

RESPONSE:

Thank you very much for pointing this out. We refrained from adding quantitative information in the general discussion to increase our manuscript’s readability. We do mention the significance levels in the method section of study 1. Moreover, as we mention in response to R.2.9, we prefer to put full statistical information in the supplements. Nevertheless, we added an additional analysis (including all statistical values) in the general discussion to underpin that our samples are comparable in their overall willingness to intervene. (p.23, line 548)” Third, some might argue that our samples differ concerning their demographics (i.e., age and gender), and that they are not representative. Although they might differ, we did not find a significant difference in their overall willingness to intervene, *t*(634) = 1.08, *p* = . 282, *d* = 0.09, 95*%* CI [-0.07, 0.24].”

**R2.12:** Incomplete Explanation of Responsibility-Based Effects: The section mentions that responsibility-based effects were not observed in more-severe situations, but it does not delve into the possible reasons for this discrepancy. Exploring potential explanations or discussing alternative theoretical frameworks that could account for the lack of responsibility effects in severe situations would enhance the analysis.

RESPONSE:

Thank you for your remark. We added a potential explanation for the discrepancy and provided an example. (p. 21 , line 480) “This pattern of results might be explained by moral values, which can predict attitudes toward victims and perpetrators. For example, a victim of sexual harassment will not be perceived as responsible and blameworthy, independent of the experimental manipulation. On the contrary, people will condemn the perpetrator for acting morally wrong, and, thus act morally courageous (68).“

**R2.13:** Insufficient Explanation of Kinship Theory Findings: Although the authors acknowledge the absence of evidence for kinship theory as a predictor, the provided explanations for this discrepancy appear somewhat speculative and lack substantial empirical support. Providing a more in-depth analysis of the potential reasons for the deviation from previous research and discussing alternative explanations would strengthen this part of the discussion.

RESPONSE:

Thank you for pointing this out. We partly replied to this comment in response to comment R2.9. Additionally, we elaborated each point of our potential explanations. (p. 22 , line 510) ”Second, we manipulated kinship rather abstract, whereas previous research used specific targets (68). To be precise, we only used close relative vs. acquaintance as categories, whereas previous studies (26,68) used concrete exemplars for close relatives (i.e., mother, father, sister, and brother). Consequently, we cannot rule out that participants may thought of moderately close relatives (i.e., aunt, uncle, grandmother, grandfather, niece, and nephew) as close ones. Third, we argue that people may have perceived those in need as co-nationals, as indicated by kinship or acquaintance relation (22,70), which in turn increases the willingness to help them as ingroup members (71,72). For example, participants could had thought of an acquaintance who supports the same sports team or of whom who attended the same concert. Therefore, they might perceived an acquaintance as in ingroup member. This argument is strengthened by the finding that prosocial behavior increases as a function of category inclusiveness (23,54,73). Fourth, although we did not assess emotional closeness, emotional closeness has been shown to account for a substantial portion of the effect of genetic relatedness on the willingness to act pro-social (68). Thus, we encourage future research to assess emotional closeness to disentangle kinship from social closeness effects.“

**R2.14:** Lack of Discussion on Sample Representativeness: The section does not mention the characteristics of the sample or any potential limitations related to sample representativeness. Including information about the demographics and characteristics of the participants, as well as addressing any potential biases or limitations associated with the sample, would add depth and context to the study's findings.

RESPONSE:

Thank you for this important remark. Indeed, our sample is not representative for non-WEIRD countries (Henrich et al., 2010), hence we added this information. (p. 23, line 548)” Third, some might argue that our samples differ concerning their demographics (i.e., age and gender), and that they are not representative. Although they might differ, we did not find a significant difference in their overall willingness to intervene, *t*(634) = 1.08, *p* = . 282, *d* = 0.09, 95*%* CI [-0.07, 0.24]. We do acknowledge that we conducted our research with WEIRD samples (81), hence our findings are limited in this way. Thus, we highly encourage future research to replicate our findings with non-WEIRD samples.“

**R2.15:** Limited Generalizability of the Findings: The section does not explicitly address the generalizability of the findings beyond the specific experimental context used in the study. Recognizing the limitations of generalizability and discussing the potential boundary conditions or contexts where the findings may not hold would provide a more comprehensive understanding of the research implications.

RESPONSE:

Thank you for pointing this out. We added more information concerning the limited generalizability of our finding. Additionally, we emphasized the importance of a representative research design. (p. 23, line 548): “Second, we only varied the moral courage scenarios; hence our sample of prosocial situations was rather limited. Moreover, we are aware of that prosociality in moral courage is complex and can occur in different facets (69). Thus, following the Brunswikian idea of systematic and representative research designs (80–82), we highly encourage future research to systematically vary situations to get a full-fledged understanding of the predictors of prosocial behavior (i.e., moral courage).”

**Reviewer #3:**

**R3.1: “**Thank you very much for giving me this opportunity to review the manuscript entitled “Standing-up against moral violations: The predicting role of attribution, kinship, and severity.", I have a few suggestions for the author(s) to incorporate in the manuscript and improve its quality further.”

RESPONSE:

First of all, we would like to thank you for your helpful and constructive feedback. Please note that, all page and line numbers refer to the “Manuscript” file.

**R3.2: “**Too complicated sentence structure that has not the capability to generalize.”

RESPONSE:

Thank you for mentioning that our sentences sometimes could have been shorter. We went over the whole manuscript and reduced the length of sentences whenever possible while improving the readability.

**R3.3: “**Results are presented in too much length that must be in a concise form.”

RESPONSE:

Thank you again, also following the suggestions of reviewer 1 and 2 we present the results as concise as possible and as comprehensive as necessary. We could move the tables to the supplements but leave this decision up to the editor.

**R3.4: “**Basic required tests are missing.”

RESPONSE:

Thank you for this remark. We tested whether gender and age interfered with our results. Both did not interfere with our results. Please see additional analysis on OSF.

**R3.5: “**Sentence is not giving complete meanings and are not well interlinked.”

RESPONSE:

Thank you for pointing out this important issue. Again, also heeding reviewer’s 1 & 2 suggestions we went over the whole manuscript and reduced the length of sentences whenever possible while improving the readability. Moreover, we aimed to improve our storytelling for a better understandability.

**R3.6: “**Revised the sentence of the entire document in complete, understandable, concise, and concrete form.”

RESPONSE:

Please see response R3.6

**R3.7: “**latest references need to be added”

RESPONSE:

Thank you for this important comment. We heeded your advice and added more recent literature. For example, we added Pfattheicher et al. (2022), and Rusch (2022) on current definitions of prosocial behavior. Moreover, we added McManus et al. (2021) on impact of relatedness on people’s intention to intervene.

**R3.8: “**The abstract and introduction are not well-written and explicitly stated.”

RESPONSE:

Again, we thank you for your helpful comment. Again, in line with comments of reviewer 2 we restructured our introduction to improve its natural flow and its readability.

**R3.9:** What is the rationale behind using underlying methods in the current research?”

RESPONSE:

Thank you very much for this comment. Indeed, we are totally aware of that we could have used different methods to address our research question. Given that we are using scenarios that would endanger participants if we would assess intervention intentions “in the wild”, we decided to use vignettes. Moreover, we opted for established manipulations and, respectively, DVs that have been successfully used in pivotal preceding research. Finally, we used an experimental design to rule out confounding variables. An effect should be present when the cause is present and that when the cause is absent, the effect should be absent also. In other words, the only way to infer causality is through comparing controlled situations: one in which the cause is present and one in which the cause is absent, (Mill, 1865). For example, in our research in condition the person in need is responsible for her/his plight (cause present), in the other condition, the person in need is no responsible for her/his plight (cause absent).

**R3.10: “**Why the authors did not employ other similar techniques and preferred this technique solely?”

RESPONSE:

Thank you for this remark. Please see our elaboration on this remark in R3.9.

**R3.11: “**There is a contribution of the study but is not well-written or explicitly stated what novelty is being added to the study.”

RESPONSE:

Thank you for pointing this out. We emphasized the novelty and contribution of our manuscript in the introduction. (p.7, line 170). “We aim to answer the question whether predictions derived from attribution and, respectively, evolutionary theory can be transferred to moral courage situations.”
