# Supplementary material for: Standing-up against moral violations: The predicting role of attribution, kinship, and severity
Source: PLoS One. 2024 Aug 12;19(8):e0307740. doi: 10.1371/journal.pone.0307740 (PMC11318881; doi:10.1371/journal.pone.0307740)
Supplement: Attachment — Submitted filename: review 02496.docx [file pone.0307740.s003.docx]

**Introduction**

The introduction provides a good overview distinguishing moral courage from helping behavior. However, it would be helpful to more clearly state the purpose and hypotheses of the current studies early on, rather than waiting until the end of the introduction.

When introducing the key predictions, explain the rationale more clearly about why responsibility attributions and kinship would interact with situation severity. Elaborate on the underlying theories and logic so the reader understands why these factors are expected to function differently across less severe vs more severe situations.

**Study 1**

In the discussion of Study 1 limitations, clarify whether all participants actually had a sibling, since assigning some only children to a "sibling condition" could create issues. Recommend changing the terminology to "close relative" in Study 2.

Provide more interpretation of the marginal kinship effect in the results and whether it suggests the predicted relationship.

Explain why moral courage may still be less severe than other emergencies and whether the findings could extend to less severe moral courage situations.

**Study 2**

The hypothesis section could state more directly that Study 2 aims to replicate Study 1 and investigate perceived costs as a mediator. Make the goals clear upfront.

Report the specific items used to measure perceived costs and provide reliability for this scale.

In the mediation analysis, explain the meaning of the direct effect results for severity on helping intentions. Does this suggest partial vs full mediation?

**General Discussion**

Summarize clearly which hypotheses were and were not supported overall across the two studies.

Provide more interpretation about why kinship effects were not found and potential reasons or moderators to investigate in future research.

Discuss whether the findings for responsibility attributions may extend to less severe moral courage situations, given the limitations noted about severity.

Conclusions could identify practical implications about encouraging moral courage and helping behaviors.
