# Supplementary material for: Standing-up against moral violations: The predicting role of attribution, kinship, and severity
Source: PLoS One. 2024 Aug 12;19(8):e0307740. doi: 10.1371/journal.pone.0307740 (PMC11318881; doi:10.1371/journal.pone.0307740)
Supplement: Attachment — Submitted filename: Response to Reviewers.docx [file pone.0307740.s004.docx]

**Editorial Comments:**

**E1:** “Reference list”

Please review your reference list to ensure that it is complete and correct.

RESPONSE:

Thank you for pointing this out. We again checked our reference list. Our refence list is complete, correct, and does not include any retracted references.

# Reviewer #1:

**R1.1:** “I commend the authors on their thoughtful response to reviews, in particular their consideration of age and sex effects both within and across the studies they present and the language they add regarding important work to follow.”

RESPONSE:

First of all, we would like to thank you again for your very helpful and constructive feedback. We address each of your comments below.

**R1.2:** " I do still have concerns regarding the operationalization of moral courage in the manuscript the degree to which it is conflated with severity in the analyses and the authors do not directly address this concern. It is not simply that moral courage situations are more severe than other scenarios they present but that they simultaneously posit moral courage as an outcome and as a predictor since it is is a level of the factor severity in their methodological design. In doing so, the methodological design and analytic approach conflates moral courage with severity such that the relationship between them cannot be empirically tested. As I stated in my original review - I don’t see this as a major issue with the analyses presented here but rather with the language/conclusions drawn by the authors. In the current version of the manuscript it is unclear whether the authors posit that varying levels of severity of a situation are important mechanisms for the prediction of moral courage or whether moral courage represents a specific level of severity.”

RESPONSE:

Thank you very much for pointing this out. Although we argue that moral courage intuitively refers to a rather high level of severity, we agree that moral courage scenarios come in different facets (Sasse et al., 2022). Especially, moral courage scenarios do vary in severity. The scenarios that we used in our MS are rather high in severity. However, we acknowledge that there are moral courage situations that might be less severe than the scenarios that we used, but still more severe than most helping scenarios. For example, speaking-up against an inappropriate joke might be less severe for an intervener than preventing someone from a physical attack (Study 1) or sexual harassment (Study 2), although more severe than lending class notes, or picking up coins, etc. Consequently, we made the point clearer in the general discussion. Additionally, we added that future research should also include full designs with moral courage/helping situations crossed by severity (less, more) to disentangle the separate effects of type of situation and severity. Thank you again for your feedback, which has helped to improve the clarity of our argument.

The section now reads (p. 21, line 474)” ). To be clear, moral courage indicates a higher level of severity.”

(p23. line 547)” Moreover, we are aware that prosocial behavior in moral courage is complex and can occur in different facets. Especially, moral courage scenarios do vary in severity (9). For example, speaking-up against an inappropriate joke might be less severe for an intervener than preventing someone from a physical attack (Study 1) or sexual harassment (Study 2), although more severe than lending class notes, or picking up coins, etc. Thus, following the Brunswikian idea of systematic and representative research designs (77–79), we highly encourage future research to systematically vary situations to get a full-fledged understanding of the predictors of prosocial behavior (i.e., moral courage). Particularly, upcoming research should orthogonally manipulate severity in moral courage scenarios.“

**R1.3: “**Finally, I noticed some issues with wording/typos in the responses that impacted the clarity of the responses. A close review of the language throughout the manuscript and edits for clarity when typos are present is needed. For example (typos/missing words highlighted with ** below):”

RESPONSE:

Thank you for your dedication to our MS. We do apologize for these inconveniences. Accordingly, we fixed our typos, added the missing words, and let proofread our MS. Thank you again!

**Reviewer #2:**

**R2.1: “**The introduction provides a good overview distinguishing moral courage from helping behavior. However, it would be helpful to more clearly state the purpose and hypotheses of the current studies early on, rather than waiting until the end of the introduction.”

RESPONSE:

First of all, we would like to thank you again for your feedback, which improved our manuscript. Please note that, all page and line numbers refer to the “Manuscript” file. We now state the purpose and hypotheses very early in the introduction.

**R2.2:** “When introducing the key predictions, explain the rationale more clearly about why responsibility attributions and kinship would interact with situation severity.”

RESPONSE:

Thank you for this comment. We added further information about our rationale of the predicted interaction (p. 7, line 166/167) “Therefore, little is known whether these findings to generalize to moral courage scenarios.“

**R2.3:** “Elaborate on the underlying theories and logic so the reader understands why these factors are expected to function differently across less severe vs more severe situations.”

RESPONSE:

Thank you for this suggestion. We elaborated on why severity should affect people’s willingness to intervene.

(p. 8, line 195) “In less-severe situations, people’s willingness to intervene is predicted to decrease, when the receiver of help is perceived to be responsible for her plight, because failing to help would have little consequences. However, we predict that people’s willingness to intervene increases when they perceive the receivers of help as being not responsible (14,16).“

(p. 9, 202) “Furthermore, we predict an opposed interaction effect between kinship and situation’s severity: in more-severe situations, people’s willingness to help a relative is higher, compared to a non-relative, but no significant effect of relationship is predicted for less-severe situations, because helping increase ultimately the prevalence of a helper’s genes (23,54).”

**R2.4** “In the discussion of Study 1 limitations, clarify whether all participants actually had a sibling, since assigning some only children to a “sibling condition” could create issues. Recommend changing the terminology “close relative” in Study 2.”

RESPONSE:

We very much agree with this comment and corrected accordingly this wording in the first revision of our MS. Moreover, we have used the term “close relative” in study 2 since the initial submission and now made sure that this is done consistently.

**R2.5** “Provide more interpretation of the marginal kinship effect in the results and whether it suggests the predicted relationship.”

RESPONSE:

Thank you for this remark. We included additional explanations in the previous version of our MS. In total, we provide five potential explanations why our studies did not show kinship effects. We derived our explanations from established theories and existing findings that are related to our work. However, we made sure that the different explanations are clearly represented. Nevertheless, we leave it up to the editor whether it’s crucial to add further explanations.

**R2.6** “Explain why moral courage may still be less severe than other emergencies and whether the findings could extend to less severe moral courage situations.”

RESPONSE:

We argue that moral courage represents a rather high level of severity. We are aware of that moral courage scenarios come in different facets (Sasse et al., 2022). Especially, moral courage scenarios do vary in severity. The scenarios that we used in our MS are rather high in severity. However, we acknowledge that there are moral courage situations that might be less severe than the scenarios that we used, but still more severe than most helping scenarios. For example, speaking-up against an inappropriate joke might be less severe for an intervener than preventing someone from a physical attack (Study 1) or sexual harassment (Study 2), although more severe than lending class notes, or picking up coins, etc. Consequently, we made to point clearer in we the general discussion. Additionally, we added that future research should also include moral courage situations that are less severe.

The section now reads (p. 23, line 547) ”Moreover, we are aware that prosocial behavior in moral courage is complex and can occur in different facets. Especially, moral courage scenarios do vary in severity (9). For example, speaking-up against an inappropriate joke might be less severe for an intervener than preventing someone from a physical attack (Study 1) or sexual harassment (Study 2), although more severe than lending class notes, or picking up coins, etc. Thus, following the Brunswikian idea of systematic and representative research designs (77–79), we highly encourage future research to systematically vary situations to get a full-fledged understanding of the predictors of prosocial behavior (i.e., moral courage). Particularly, upcoming research should orthogonally manipulate severity in moral courage scenarios.“

**R2.7** “The hypothesis section could state more directly that Study 2 aims to replicate Study 1 and investigate perceived costs as a mediator. Make the goals clear upfront.“

RESPONSE:

Thank you for pointing this out. In line with your recommendation, the first sentence of Study 2 now reads: “Study 2 aimed to replicate Study 1, and to examine costs for helping as an underlying psychological process.

**R2.8:** “Report the specific items used to measure perceived costs and provide reliability for this scale.”

RESPONSE:

We agree with this comment as this information was already included in the first version of our MS.

**R2.9:** “In the mediation analysis, explain the meaning of the direct effect results for severity on helping intentions. Does this suggest partial vs full mediation?“

RESPONSE:

Again, we appreciate this comment and checked that the information about the mediation that we included in the revised manuscript is clearly represented.

**R2.10:** “Provide more interpretation about why kinship effects were not found and potential reasons or moderators to investigate in future research.”

RESPONSE:

Please see response to comment R2.5

**R2.11:** “Discuss whether the findings for responsibility attributions may extend to less severe moral courage situations, given the limitations noted about severity.”

RESPONSE:

Thank you for pointing this out. Please see our response R2.6

**R2.12:** “Conclusions could identify practical implications about encouraging moral courage and helping behaviors.”

RESPONSE:

Thank you for your remark. We added a potential practical implication of our findings (p. 24, line 569). “In addition to our theoretical contributions, our findings can be applied to improve training programs that foster prosocial behavior. Especially, our results can help to develop tailored exercises that aim to improve moral courage.”
